# 

**Published:** 1889-09

**Authors:** 


### CONTENTS:

Article I.—National Association of Dental Examiners. . 193
" II.—National Association of Dental Faculties. . 197
" III.—Filling Proximal Cavities in Bicuspids and Molars. 201
" IV.—The Micro-Organisms of the Mouth and their Association with Disease. By Geo. W. Watson. 212
" V.—Escharotics and Coagulants. By Dr. A. W. Harlan. . . . . . . 217
" VI.—A Few Facts about Platina. . . . 225
" VII.—Campho Phenique By J. Foster Flagg, D. D. S. 229
" VIII.—Some Incidents of Practice in Mexico. By Dr. N. H. Wheeler. . . . 231
The Dental Protective Association of the United States. . 232

### EDITORIAL.

The University of Maryland Dental Department . . 238
The Dental Meetings at Saratoga. . . . . . 239

